# Screening inflammatory protein biomarkers on premature infants with necrotizing enterocolitis

**DOI:** 10.1007/s00011-023-01702-6

**Published:** 2023-02-18

**Authors:** Huifang Dong, Lingling Zhang, Bingbing Li, Jing Li, Yanshan Chen, Seidu A. Richard, Yiran Xu, Changlian Zhu

**Affiliations:** 1 Henan Pediatric Clinical Research Center and Henan Key Laboratory of Child Brain Injury, Third Affiliated Hospital and Institute of Neuroscience, Zhengzhou University, Zhengzhou, 450052 China; 2 Department of Neonatology, Third Affiliated Hospital of Zhengzhou University, Zhengzhou, 450052 China; 3 Department of Women’s and Children’s Health, Karolinska Institute, Stockholm, Sweden; 4 Center for Brain Repair and Rehabilitation, Institute of Neuroscience and Physiology, University of Gothenburg, 40530 Goteborg, Sweden

**Keywords:** Biomarker, Inflammation, Necrotizing enterocolitis, OLINK, Proteomics

## Abstract

**Objective:**

This study aimed to explore potential inflammatory biomarkers for early prediction of necrotizing enterocolitis (NEC) in premature infants.

**Methods:**

Plasma samples were collected from premature infants with NEC (*n* = 30), sepsis (*n* = 29), and controls without infection (*n* = 29). The 92 inflammatory-related proteins were assessed via high-throughput OLINK proteomics platform**.**

**Results:**

There were 11 inflammatory proteins that significate differences (*p* < 0.05) among NEC, sepsis and control preterm infants, which include IL-8, TRAIL, IL-24, MMP-10, CCL20, CXCL1, OPG, TSLP, MCP-4, TNFSF14 and LIF. A combination of these 11 proteins could serve as differential diagnosis between NEC and control infants (AUC = 0.972), or between NEC and sepsis infants (AUC = 0.881). Furthermore, the combination of IL-8, OPG, MCP-4, IL-24, LIF and CCL20 could distinguish Stage II and III of NEC (AUC = 0.977). Further analysis showed the combination of IL-8, IL-24 and CCL20 have the best prediction value for NEC and control (AUC = 0.947), NEC and sepsis (AUC = 0.838) and different severity of NEC (AUC = 0.842).

**Conclusion:**

Inflammatory proteins were different expressed in premature infants with NEC compared with controls or sepsis. Combining these proteins provide a higher diagnostic potential for preterm NEC infants.

**Supplementary Information:**

The online version contains supplementary material available at 10.1007/s00011-023-01702-6.

## Introduction

Necrotizing enterocolitis (NEC) is a serious gastrointestinal disease of newborn with an incidence of 5–13% [1], and it is also one of the important causes of death in premature infants with a mortality rate of 20–30% [2, 3]. It is mainly seen in premature infants, and about 30–50% of infants with NEC need surgical treatment [4–6]. The mortality rate of premature infants with NEC who need surgical treatment is up to 50% [4, 7].

The causes of NEC are multifactorial, and premature delivery, infection, asphyxia and formula feeding are considered the main risk factors [8, 9]. The occurrence of NEC is related to intestinal flora disorder, intestinal microvascular tension, gene susceptibility and other factors [10, 11]. However, infection and inflammation have been agreed to play crucial role in the initiation and process of NEC and NEC is generally regarded as an infectious or inflammatory disease [12, 13]. The pathophysiological basis is still not completely clear, and it is difficult to diagnose or distinguish from sepsis in the early stages and current infection makers such as C-reactive protein (CRP) and procalcitonin are not specific [14].

NEC is still with high mortality and early diagnosis and intervention could reduce the mortality and improve the progress [15, 16]. Thus, it is important to screen and identify specific NEC biomarkers to be used for early clinical diagnosis and differential diagnosis. We hypothesized that infection and inflammation-related enterocolitis could induce organ-related inflammation markers. In this study, we used both health and sepsis as control groups to compare with NEC in order to screen the circulating inflammatory biomarkers of NEC in preterm infants using the OLINK (antibody-based proximity extension assay platform) technology.

## Methods

### Patient recruitment

We recruited 30 cases of preterm infants with NEC in the Third Affiliated Hospital of Zhengzhou University from 2018.01 to 2021.06, the diagnosis and stage of NEC were established according to modified Bell’s staging, consisting of the severity of systemic, intestinal, radiographic, and laboratory findings. The inclusion criteria were gestational age < 37 weeks, birth weight < 2500 g and Bell’s stage II or III. The exclusion criteria were infants with major anomalies such as chromosomal or genetic anomalies, cyanotic heart defects, gastrointestinal abnormalities or other malformations requiring surgical correction during the neonatal period, or with infectious diseases such as sepsis and pneumonia within one week before the onset of NEC. Stage II was defined as NEC with signs such as pneumatosis intestinalis, ascites, and tenderness of the abdomen. Stage III was defined as NEC with signs of peritonitis and intestinal perforation. Patients diagnosed with NEC who did not receive surgical therapy were defined as medical NEC patients, and patients who received surgical treatment were defined as surgical NEC patients. Among the 30 NEC infants, the onset of symptoms included: abdominal distension in 12 cases (40%), hematochezia in 10 cases (33.3%), fever in 4 cases (13.3%), and feeding intolerance in 4 cases (13.3%). The first abdominal radiography was done within 12 h for infants with the above symptoms, and 16 cases (53.3%) showed intestinal dilatation, 8 cases (26.7%) showed widening of intestinal space/fixation of intestinal loops, and only 6 cases (20%) showed pneumatized intestinal wall/portal venous.

In addition, 29 preterm infants with sepsis admitted in our hospital in the same period were selected as the sepsis group. Their gestational ages (± 1 week), birth weights (± 200 g) and onset ages (± 5 days) were matched with NEC group. Clinical diagnosis was founded on abnormal clinical manifestation, and any of the following conditions simultaneously: ① blood non-specific examination ≥ 2 items are positive, ② cerebrospinal fluid examination shows purulent meningitis change, and ③ pathogenic bacteria DNA or antigen detected in blood. Non-specific blood tests consisted of ① white blood cell (WBC) < 5 *10^9^/L or > 20*10^9^/L, ② CRP ≥ 10 mg/L, ③ procalcitonin > 0.5 mg/L, and ④ platelet (PLT) < 100*10^9^/L. Thus, definitive diagnosis was established using clinical manifestations and positive results of blood cultures or cerebrovascular fluid cultures and not gastrointestinal symptoms such as abdominal distension, diarrhea or bloody stool at the onset. Among the 29 infants, the onset symptoms were skin blanching in 9 cases (31.0%), fever in 8 cases (27.6%), apnea in 6 cases (20.7%), and poor response in 6 cases (20.7%).

Moreover, another 29 premature infants at the same period without NEC or infectious diseases were selected as healthy control group (HC group). Their gestational ages and birth weights were matched with the NEC group. The exclusion criteria of the sepsis group and control group were infants with major anomalies.

### Specimen collection

Blood sample of 200 µl with heparin-anticoagulated was collected within 12 h after the onset of NEC or sepsis, and the corresponding ages after birth in the HC group. If blood transfusion or surgery was needed, the blood samples were collected before these treatment modalities. The plasma was separated by centrifugation (1000*g* × 5 min) at 4 °C and stored at − 80 °C freezer. This study was approved by the Ethics Committee of the Third Affiliated Hospital of Zhengzhou University (2018-04). In addition, written informed consents were obtained from the parents of all the infants who participated in this study.

### OLINK assay

Protein was measured using the OLINK 92 panel (OLINK Proteomics AB, Uppsala, Sweden) according to the manufacturer’s instructions. The Proximity Extension Assay (PEA) technology used for the OLINK protocol has been well described [17], and enables 92 analytes to be analyzed simultaneously using 1 µl of plasma. In brief, pairs of oligonucleotide-labeled antibody probes bind to targeted proteins, and if the two probes are brought in close proximity the oligonucleotides will hybridize in a pair-wise manner. The addition of a DNA polymerase leads to a proximity-dependent DNA polymerization event, generating a unique PCR target sequence. The resulting DNA sequence was subsequently detected and quantified using a microfluidic real-time PCR instrument (Signature Q100, OLINK). The resulting Ct-data is then quality controlled and normalized using a set of internal and external controls. The final assay read-out was presented in Normalized Protein eXpression (NPX) values, which is an arbitrary unit on a log2-scale where a high value corresponds to a higher protein expression.

### Statistics and multivariate data analysis

Normally distributed data were presented as mean ± standard deviation and non-parametrically data were presented as median (interquartile range, IQR). Qualitative data were summarized into percentages. The Chi-square, Fisher exact, Mann–Whitney *U*, and Kruskal–Wallis tests were used to compare the demographic data, clinical characteristics, and inflammation proteins concentrations among groups, where appropriate. Inflammation proteins concentrations were all logarithm (2)-transformed for analysis.

Univariate and multivariable logistic regressions were used to investigate the association between inflammation proteins levels and risk of NEC and compared with control or sepsis groups. Discrimination capacities were determined by calculating the area under the receiver-operating characteristic curve (AUC) and the most appropriate cutoff value was determined based on the Youden index.

The statistical analysis was performed using R statistical software and SPSS 23.0 software (IBM, Armonk, USA). *p* < 0.05 were accepted as statistically significant.

## Results

### Population characteristics

Stratification of the 30 premature infants with NEC revealed that 18 cases (60%) were at stage II and 12 cases (40%) were at stage III. In addition, 19 cases (63.3%) were medical NEC and 11 cases (36.7%) were surgical NEC. Among the 11 surgical NEC cases, 7 cases (63.6%) had terminal ileum or ileocecal necrosis, including 1 case of perforated terminal ileum, 2 cases had middle intestinal necrosis (18.2%), 1 case had total intestinal necrosis (9.1%) and 1 case had colon necrosis (9.1%). Typical inflammatory changes were observed under the light microscope after HE staining in the necrotic intestinal tissues.

For the sepsis, 8 cases (27.6%) were clinically diagnosed and 21 cases (72.4%) were diagnosed with laboratory investigations. Out of the 21 cases established with laboratory investigation, 14 cases (66.7%) had Gram-negative infections, 5 cases (23.8%) had Gram-positive infections and 2 cases (9.5%) had fungal infections in the sepsis group. The basic characteristics had no differences among the 3 groups except high mortality in NEC group (Table 1).

**Table 1:** Population characteristics

Characteristics	NEC group (*n* = 30)	Sepsis group (*n* = 29)	HC group (*n* = 29)	*p* value
Gestational age (weeks)	33.24 ± 2.12	32.81 ± 2.54	32.81 ± 2.03	0.702
Body weight at birth (g)	1640 (1345, 2107.5)	1450 (1100, 1890)	1570 (1450, 1920)	0.186
Male, no. (%)	17 (56.7)	21 (72.4)	18 (62.1)	0.444
Singleton, no. (%)	20 (66.7)	23 (79.3)	22 (75.9)	0.519
Natural pregnancy, no. (%)	26 (86.7)	24 (82.8)	25 (86.2)	0.832
Method of delivery, no. (%)				
Vaginal delivery, no. (%)	3 (10.0)	7 (24.1)	7 (24.1)	0.281
Cesarean section, no. (%)	27 (90.0)	22 (75.9)	22 (75.9)	0.281
Apgar score				
Apgar score at 1 min	9.0 (8.0, 9.0)	8.0 (7.5, 9.0)	9.0 (7.0, 9.0)	0.660
Apgar score at 5 min	10 (9.0, 10)	9.0 (9.0, 10)	10 (8.5, 10)	0.274
Days of onset, (d)	17.10 ± 9.24	16.90 ± 14.12	15.52 ± 6.98	0.491
Complication, no. (%)				
RDS	18 (60)	18 (62.1)	23 (79.3)	0.226
IVH grade ≥ III	2 (6.7)	4 (13.8)	2 (6.9)	0.652
PVL	1 (3.3)	2 (6.9)	1 (3.4)	0.843
CHD	22 (73.3)	21 (72.4)	23 (79.3)	0.863
BPD	2 (6.7)	3 (10.3)	1 (3.4)	0.691
SGA, no. (%)	3 (10)	4 (13.8)	4 (13.8)	0.851
Unclosed PDA, no. (%)	4 (13.3)	5 (17.2)	3 (10.3)	0.767
Maternal age at delivery (years)	31.5 ± 5.14	28.97 ± 4.96	30.45 ± 5.73	0.187
Antenatal corticosteroids, no. (%)	21 (70)	22 (75.9)	23 (79.3)	0.705
Antenatal complications, no. (%)				
HDP	9 (30)	10 (34.5)	9 (31)	0.959
GDM	7 (23.3)	5 (17.2)	4 (13.8)	0.629
PROM	7 (23.3)	4 (13.8)	7 (24.1)	0.553
Threatened preterm	5 (16.7)	6 (20.7)	4 (13.8)	0.831
Others	9 (30.0)	10 (34.5)	12 (41.4)	0.445
Outcome, no. (%)				
Recovery	27 (90)	28 (96.6)	29 (100)	0.493
Death	3 (10.0)	1 (3.4)	0	< 0.001 ***

Data expressed as mean ± standard deviation or percentage. Apgar score expressed as interquartile range. IL-8 (interleukin-8), TRAIL (TNF-related apoptosis-inducing ligand), IL-24 (interleukin-24), MMP-10 (matrix metalloproteinase-10), CCL20 (C–C motif chemokine 20), CXCL1 (C–X–C motif chemokine 1), OPG (osteoprotegerin), TSLP (Thymic stromal lymphopoietin), MCP-4 (monocyte chemotactic protein-4),TNFSF14 (tumor necrosis factor ligand superfamily member 14), LIF (Leukemia inhibitory factor)

*RDS* respiratory distress syndrome, *IVH* intraventricular hemorrhage, *PVL* periventricular leukomalacia, *CHD* congenital heart disease, *BPD* bronchopulmonary dysplasia, *SGA* small for gestational age, *PDA* patent ductus arteriosus, *HDP* hypertensive disorders of pregnancy, *GDM* gestational diabetes mellitus, *PROM* premature rupture of membrane

*p < 0.05, **p < 0.01, ***p < 0.001

### OLINK proteomics assay

**Fig. 1:**
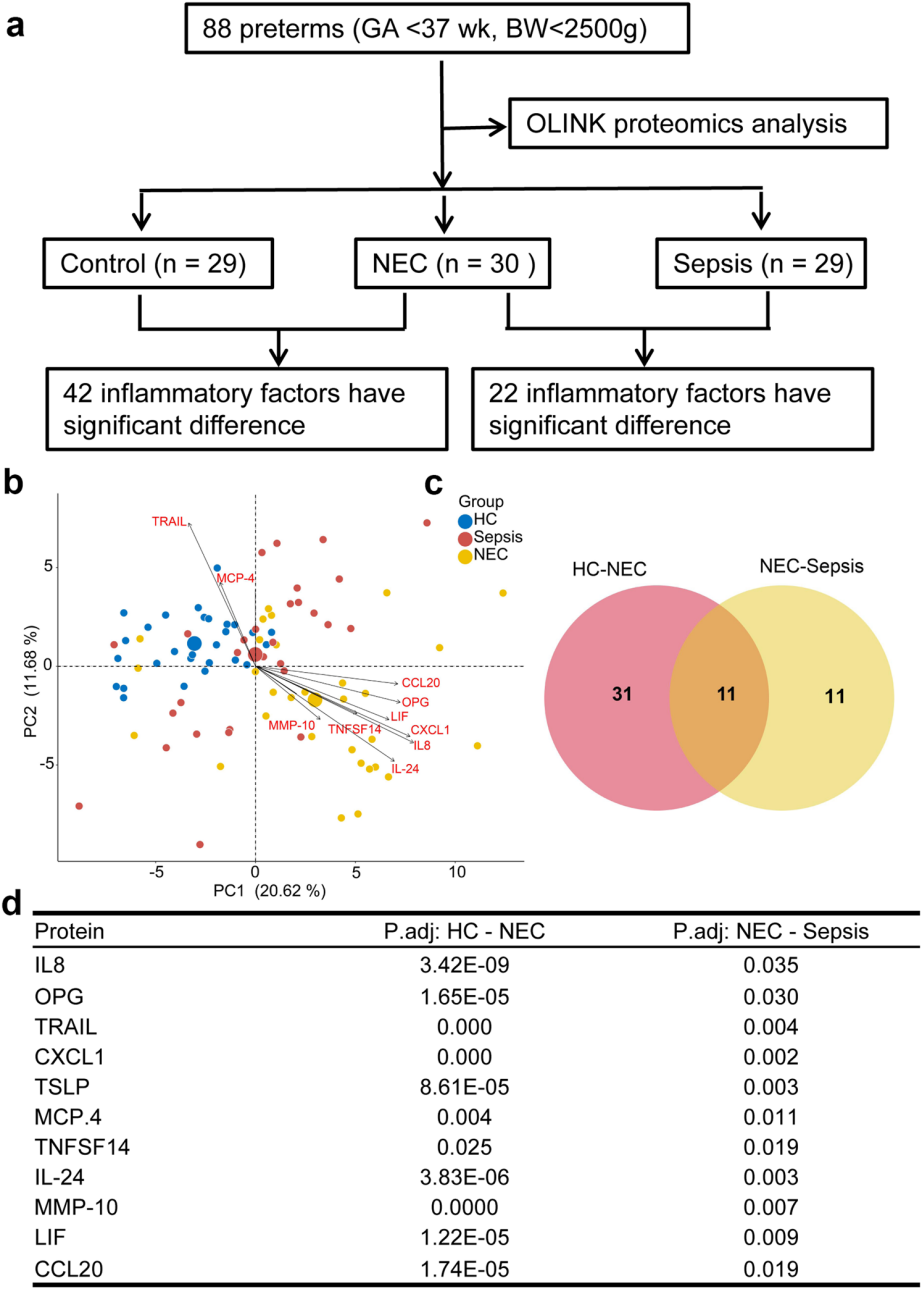
The flow chart of the study and the characteristics of proteins detected by OLINK in NEC, sepsis and HC groups. **A** The flow chart of the study cohort. **B** Principal component analysis (PCA) based on proteins in NEC, sepsis and HC groups. **C** Wayne diagram showing the common and unique proteins in NEC and sepsis groups. **D** Comparison of differentially expressed proteins in HC vs NEC and NEC vs sepsis groups

**Fig. 2:**
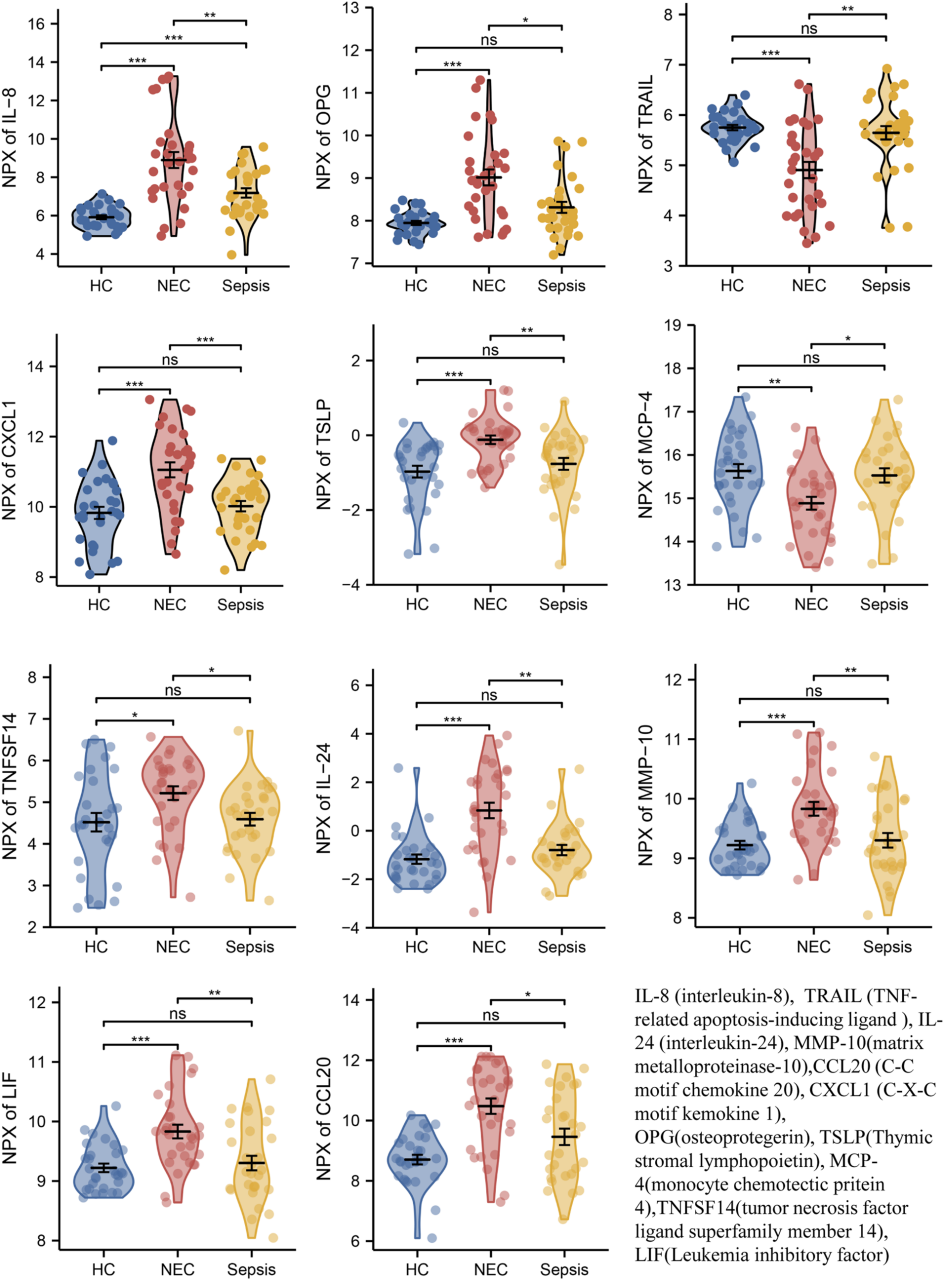
Expression profile of co-differential proteins in three cohorts. Violin plot showing the differences in co-differential proteins between the three groups. NPX, normalized protein expression, is OLINK’s arbitrary unit which is in Log2 scale. It is calculated from Ct values and data pre-processing (normalization) is performed to minimize both intra- and inter-assay variation

All the 92 plasma proteins were identified in the three groups, and 42 of them differed significantly between NEC and HC groups, 22 proteins differed significantly between NEC and sepsis groups, and overall 11 proteins differed significantly among the three groups which could be used as protein biomarkers (Fig. 1). Moreover, proteins such as TNF-related apoptosis-inducing ligand (TRAIL) and monocyte chemotactic protein-4 (MCP-4) were lower in NEC group, while the rest of other proteins were significantly higher (Fig. 2). These results indicate that infection and site of the infection may to some extent determine the inflammation-associated protein expressions in NEC.

### NEC and healthy control

Logistic regression analysis with co-founding factors such as gestational ages, birth weights, sex and fetal distress in the NEC and HC groups revealed that 11 inflammatory proteins potentially distinguished NEC from HC groups. In addition, all the 11 proteins remained significantly different after statistical adjustments (Supplementary Table 1). Furthermore, interleukin-8 (IL-8, AUC = 0.907), C–C motif chemokine 20 (CCL20, AUC = 0.851), leukemia inhibitory factor (LIF, AUC = 0.846), interleukin-24 (IL-24, AUC = 0.844), osteoprotegerin (OPG, AUC = 0.830), and thymic stromal lymphopoietin (TSLP, AUC = 0.814) had higher diagnostic values than CRP (AUC = 0.800). Nevertheless, a combination of these 11 protein biomarkers had significantly improved (AUC = 0.972) diagnostic value than the individuals (Table 2, Supplementary Fig. 1).

**Table 2:** The diagnostic value of protein biomarkers and WBC, N%, PLT and CRP between NEC and HC group

Biomarkers	Cutoff value	Sensitivity	Specificity	AUC (95% CI)	*p* value
IL-8	6.870	0.966	0.867	0.907 (0.813–1.0)	< 0.001***
OPG	8.508	1.0	0.667	0.830 (0.713–0.947)	< 0.001***
TRAIL	5.279	0.966	0.667	0.793 (0.666–0.920)	< 0.001***
CXCL1	11.023	0.931	0.567	0.787 (0.670–0.905)	< 0.001***
TSLP	− 0.177	0.966	0.633	0.814 (0.702–0.926)	< 0.001***
MCP-4	15.661	0.517	0.900	0.737 (0.608–0.865)	0.002**
TNFSF14	5.024	0.724	0.700	0.675 (0.531–0.818)	0.021*
IL-24	− 0.147	0.897	0.733	0.844 (0.737–0.950)	< 0.001***
MMP-10	9.240	0.621	0.900	0.799 (0.682–0.916)	< 0.001***
LIF	− 1.141	0.793	0.767	0.846 (0.748–0.944)	< 0.001***
CCL20	9.994	0.966	0.667	0.851 (0.745–0.956)	< 0.001***
WBC^&^	8.270	0.862	0.633	0.720 (0.580–0.860)	0.004**
N%^&^	58.850	0.966	0.600	0.730 (0.585–0.876)	0.002**
PLT^&^	241.50	0.793	0.667	0.682 (0.540–0.824)	0.016*
CRP^&^	12.530	1.0	0.800	0.917 (0.839–0.994)	< 0.001***
Model1^#^	–	0.966	0.900	0.972 (0.940–1.00)	< 0.001***

*WBC* white blood cell, *N* neutrophil, *Hb* hemoglobin, *PLT* platelet, *CRP* C-reactive protein

*p < 0.05, **p < 0.01, ***p < 0.001

^&^The routine blood test time was within 12 h after onset of NEC or the corresponding time of HC groups

^#^Model 1 indicates the combination of the above 11 selected cytokines

### NEC and sepsis

**Fig. 3:**
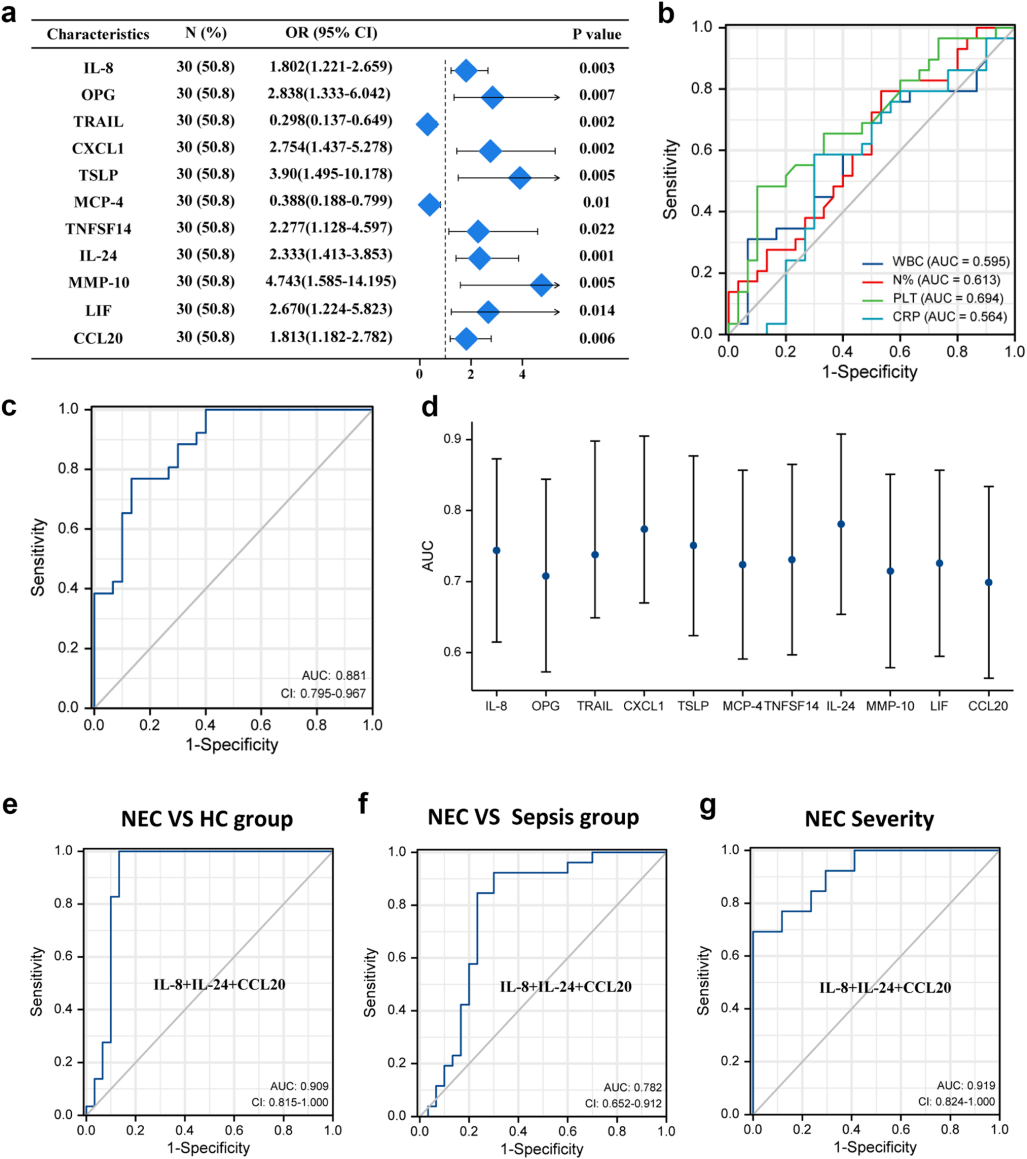
ROC curve of proteins in NEC and sepsis groups. **a** Diagnostic efficacy of co-expressed proteins in distinguishing NEC from sepsis. OR = odds ratio. **b** ROC curve analysis of WBC, N%, PLT, CRP in NEC and sepsis group, WBC: white blood cell, N%: neutrophil percentage, PLT: platelet, CRP: C-reactive protein. **c** ROC curve analysis of the combination of the 11 selected proteins. **d** Characteristics of AUC of co-differential proteins in NEC and sepsis group. AUC: Area under the curve. **e** ROC curve analysis of IL-8 + IL-24 + CCL20 in NEC and HC group. **f** ROC curve analysis of IL-8 + IL-24 + CCL20 in NEC and sepsis group. **g** ROC curve analysis of IL-8 + IL-24 + CCL20 in different severity of NEC

Similarly, logistic regression analysis with co-founding factors such as gestational ages and birth weights in the NEC and sepsis groups revealed that 11 proteins potentially distinguished NEC and sepsis groups as observed in the NEC and HC groups. All the 11 proteins remained significantly different after statistical adjustments (Supplementary Table 2). However, CRP (AUC = 0.564) had a low diagnostic value for NEC, while IL-24 (AUC = 0.781), C–X–C motif chemokine 1 (CXCL1, (AUC = 0.774)), TSLP (AUC = 0.751) and IL-8 (AUC = 0.744) had good diagnostic values for NEC. Moreover, an improved diagnostic value (AUC = 0.881) was observed when all the 11 biomarkers were combined (Table 3, Fig. 3a–d, Supplementary Fig. 2).

**Table 3:** The diagnostic value of protein biomarkers and WBC, N%, PLT and CRP between NEC and sepsis group

Biomarkers	Cutoff value	Sensitivity	Specificity	AUC (95% CI)	*p* value
IL-8	8.786	0.897	0.533	0.744 (0.615–0.873)	0.001***
OPG	8.782	0.828	0.60	0.708 (0.573–0.844)	0.006**
TRAIL	5.414	0.828	0.70	0.738 (0.649–0.898)	0.002**
CXCL1	10.511	0.793	0.733	0.774 (0.670–0.905)	< 0.001***
TSLP	− 0.213	0.793	0.667	0.751 (0.624–0.877)	0.001**
MCP-4	15.354	0.690	0.733	0.724 (0.591–0.857)	0.003**
TNFSF14	5.422	0.931	0.533	0.731 (0.597–0.865)	0.002**
IL-24	0.113	0.885	0.667	0.781 (0.654–0.908)	< 0.001***
MMP-10	9.229	0.552	0.900	0.715 (0.579–0.851)	0.005**
LIF	− 0.734	0.862	0.600	0.726 (0.595–0.857)	0.003**
CCL20	10.421	0.724	0.600	0.699 (0.564–0.834)	0.009**
WBC^&^	15.25	0.310	0.933	0.595 (0.446–0.743)	0.211
N%^&^	70.65	0.793	0.467	0.613 (0.468–0.758)	0.135
PLT^&^	123.5	0.483	0.900	0.694 (0.558–0.831)	0.010*
CRP^&^	24.92	0.586	0.70	0.564 (0.411–0.717)	0.396
Model1^#^	–	0.769	0.867	0.881 (0.795–0.967)	< 0.001***

*p < 0.05, **p < 0.01, ***p < 0.001

^&^The routine blood test time was within 12 h after onset of NEC or sepsis

^#^Model 1 indicates the combination of the above 11 selected cytokines

### Severity of NEC and protein expression

Plasma protein biomarkers such as IL-8, OPG, MCP-4, IL-24, LIF, CCL20 and PLT correlated well with the severity of NEC. In addition, MCP-4 and PLT levels were lower in stage III of infants with NEC compared to that of stage II, while the expression levels of the other protein biomarkers were higher in the stage III than that of in stage II (Table 4). Furthermore, CCL20 (AUC = 0.887), OPG (AUC = 0.851), PLT (AUC = 0.808), IL-8 (AUC = 0.778), and IL-24 (AUC = 0.735) had good diagnostic values in determining the severity of NEC. However, when 6 differentially expressed biomarkers include IL-8, OPG, MCP-4, IL-24, LIF and CCL20 were combined, their diagnostic value significantly increased (AUC = 0.977) (Table 5 and Supplementary Fig. 3).

**Table 4:** Protein biomarkers and WBC, N%, PLT and CRP in different severity of NEC

Biomarkers (NPX)	Stage II (*n* = 18)	Stage III (*n* = 12)	*p* value
IL-8	7.816 ± 1.531	10.514 ± 2.299	0.001**
OPG	8.447 ± 0.640	9.870 ± 0.873	< 0.001***
TRAIL	5.07 ± 0.795	4.65 ± 0.982	0.205
CXCL1	10.80 ± 0.987	11.43 ± 1.342	0.145
TSLP	− 0.17 ± 0.679	− 0.04 ± 0.570	0.571
MCP-4	15.21 ± 0.784	14.40 ± 0.591	0.005**
TNFSF14	5.30 ± 0.961	5.10 ± 0.821	0.555
IL-24	0.11 ± 1.634	1.93 ± 1.331	0.003**
MMP-10	9.71 ± 0.612	10.02 ± 0.635	0.186
LIF	− 0.56 ± 1.180	1.13 ± 2.879	0.033*
CCL20	9.81 ± 1.355	11.48 ± 0.704	0.001**
WBC^&^	7.690 (5.322, 10.795)	6.775 (4.22, 9.818)	0.545
N%^&^	54.3 (32.325, 73.650)	72.55 (64.95, 75.28)	0.059
PLT^&^	248 (195.5, 397.25)	157.5 (76.75, 222.5)	0.004**
CRP^&^	29.345 (3.18, 76.035)	53.57 (32.838, 116.97)	0.095

Date expressed as mean ± standard deviation

*NPX* normalized protein expression

*p < 0.05, **p < 0.01, ***p < 0.001

^&^The routine blood test time was within 12 h after onset of NEC

**Table 5:** The biomarkers between stage II and stage III of NEC

Biomarkers	Cutoff value	Sensitivity	Specificity	AUC (95% CI)	*p* value
IL-8	9.334	0.615	0.882	0.778 (0.601–0.955)	0.003**
OPG	9.365	0.615	0.941	0.851 (0.712–0.990)	< 0.001***
MCP-4	15.50	0.923	0.353	0.690 (0.512–0.868)	0.005**
IL-24	1.50	0.615	0.765	0.735 (0.552–0.918)	0.004**
LIF	0.500	0.385	0.824	0.674 (0.487–0.862)	0.020*
CCL20	11.50	0.615	1.0	0.887 (0.775–0.999)	0.001**
PLT	237.5	0.917	0.556	0.808 (0.645–0.970)	0.005**
Model1^#^	–	0.923	1.0	0.977 (0.930–1.0)	< 0.001***

*p < 0.05, **p < 0.01, ***p < 0.001

^#^Model 1 indicates the combination of the above 6 selected cytokines including IL-8, OPG, MCP-4, IL-24, LIF, and CCL20

### Combination of NEC and HC as well as NEC and sepsis

It worth noting that IL-8 and IL-24 were both differentially expressed between NEC and HC as well as NEC and sepsis, and between different severities of NEC. A combination of IL-8, IL-24, and CCL20 had best predictive values in distinguishing NEC and HC, NEC and sepsis, and different severities of NEC with corresponding AUCs of 0.909, 0.782, and 0.919, respectively, when the predictive values of the combinations of IL-8 and IL-24 with each of other biomarkers were assessed to facilitate clinical application (Fig. 3e–g). However, the combination of IL-8, IL-24, and CCL20 still showed a lower predictive value than the combination of all the 11 or 6 protein biomarkers.

## Discussion

In this study, we used high-throughput OLINK proteomics analysis technology and simultaneous analysis of inflammatory proteins with minimal samples to screen high specificity and sensitivity biomarkers for NEC by comparing with preterm control and sepsis. We selected 11 inflammation proteins which belong to interleukin family (IL-8, IL-24, TSLP, and LIF), the tumor necrosis factor family [OPG, TRAIL, and tumor necrosis factor ligand superfamily member 14 (TNFSF14)], and the chemokine family [CCL20, MCP-4, CXCL1, matrix metalloproteinase-10 (MMP-10)]. Most of these 11 inflammatory protein biomarkers are capable of distinguishing NEC and HC, but the combination of these markers could achieve a much better diagnostic value than individual inflammatory biomarkers and CRP, which is the most used and economic indicator to determine the presence or absence of infections in clinical practice. We demonstrated that combination of IL-8, IL-24 and CCL20 have the best value to identify NEC from sepsis or the severity of NEC.

CRP has been widely used clinically for aiding the diagnosis of bacterial infection by combining with WBC and neutrophil accounts in blood routine test [14]. However, these markers are neither organ infection specific nor bacteria specific, which makes more complicated for preterm infants because of lacking obvious symptoms or signs after bacterial infection [18]. Our results showed even though CRP has a high value to identify NEC from control, but with limited value to distinguish NEC and sepsis, which could be related with that NEC is easy to induce sepsis.

We found IL-24, CXCL1, TSLP and IL-8 were significantly different between the NEC and sepsis groups. The analysis of the groups on the severity of NEC showed that CCL20, OPG, PLT, IL-8, and IL-24 were significantly related to the severity of the disease. Among these biomarkers, IL-8 and IL-24 were significantly different between NEC and sepsis and between different severities of NEC, which indicates that IL-8 and IL-24 levels have better clinical application value in distinguish NEC from sepsis or in distinguish the severity of NEC.

IL-8 is a major chemokine of neutrophils and lymphocytes that is capable of inducing T cell migration and participation in the regulation of various inflammatory responses [19]. Many studies have confirmed that the IL-8 levels in children with NEC were significantly higher than those in children who underwent surgery due to other diseases and healthy newborns [20–25], and the IL-8 level was positive related with the severity of NEC [26–28]. This study confirmed that IL-8 was increased in NEC and more pronounced in the severe NEC infants. Furthermore, IL-8 levels in NEC infants were significantly higher than those of the infants with sepsis, which suggests that IL-8 not only help to diagnosis NEC, but also to distinguish NEC and sepsis.

IL-24 is a member of the IL-10 family of cytokines and is expressed by monocytes and T lymphocytes (mainly Th2) [29], which has proinflammatory and proapoptotic effects and is associated with inflammation and autoimmune diseases. In patients with inflammatory bowel disease, IL-24 is capable of inducing the expression of proinflammatory cytokines such as TNF-α and IL-6 as well as induction of inflammation and immune cell infiltration during tissue damage [30]. Some studies reported changes of IL-24 in intestinal disease [31, 32], and no studies have investigated the relationship between IL-24 and the occurrence of NEC. To best of our knowledge, this is the first to report the diagnostic value of IL-24 elevation in differentiating NEC and sepsis and in determining the severity of NEC. However, whether IL-24 plays a proinflammatory or anti-inflammatory role in the pathogenesis of NEC remains to be investigated further.

OPG is produced by B cells, macrophages, dendritic cells, intestinal epithelial cells, and mediates a variety of cellular functions in the immune system [33]. Existing studies have shown that OPG participates in a complex cytokine network and may become a new marker of intestinal inflammation [34, 35]. It has been reported that intestinal epithelial cells could produce OPG, which could stimulate the secretion of IL-8 by the epithelial cells and also promote the inflammatory response as well as destroy the integrity and function of the intestinal epithelial tight junction barrier together with TNF-α. It is speculated that OPG and TNF-α have similar proinflammatory effects. We found that the OPG level was significantly different between the NEC stage III and II, which indicates that OPG is related to the severity of intestinal injury.

CCL20 is expressed by helper T lymphocytes (Th17 cells) and DCs and has a strong chemotactic effect. It is induced in epithelial cells in vitro by lipopolysaccharide and inflammatory cytokines such as TNF-α and interferon-γ [36]. CCL20 is predominantly expressed in vivo by the intestinal epithelium, especially in response to inflammatory stimuli [37, 38], and the level of CCL20 was positive correlated with the degree of inflammation. We found there was a significant difference in CCL20 between NEC infants with different severities (AUC = 0.887), which indicates that CCL20 can be used to assess the severity of NEC.

TSLP is an IL-7-like cytokine that is mainly expressed in human epithelial cells and keratinocytes. Intestinal epithelial cells (IECs) and DCs may also express TSLP and maintain intestinal immune balance through barrier function and active regulation of intestinal immune responses [39, 40]. Clinical studies found that the mRNA expression level of TSLP was significantly lower in ulcerative colitis patients than in the control group, and the low expression of TSLP showed a positive correlation with the severity of ulcerative colitis [41, 42]. Up to now, there is no research on the role of TSLP in the development of NEC, and we found TSLP level was significantly different between the NEC and sepsis, which suggests that TSLP may plays an important role in the pathophysiology of NEC.

This study is the first study to investigate the plasma levels of 92 inflammatory proteins in preterm infants with NEC using a novel target proteomics approach based on proximity extension assay technology. Some biomarkers have not been reported in previous studies, which may provide new ideas for future research. We compared NEC and sepsis, the two infectious diseases, but not just compare the difference between NEC and the control group of non-infectious diseases. It is capable of showing the difference in inflammatory factors between intestinal infection and non-intestinal infection, so as to predict NEC in the early stage of disease, which has good clinical value.

It should be noted that many studies found that 85% of NEC infants in high-income countries occurred at gestational ages < 32 weeks or birth weights < 1500 g [3, 6, 43, 44]. The average gestational age of preterm infants employed in this study cohort was 33.2 ± 2.1 weeks, which was slightly higher than what was reported in high-income country [45]. This is exclusively due to the influence of gestational age, birth weight and the onset (days) of NEC or sepsis after birth among NEC, sepsis and control groups. However, our previous study cohort showed an average gestational age of 30.3 ± 2.1 weeks and birth weight of 1166 ± 203 g with 166 NEC cases [11]. In general, the gestational ages of NEC infants in the study were similar to the report from high-income countries [45].

There are some limitations in this study. First, inflammatory proteins produced by local intestinal tissue will enter blood circulation in intestinal infection diseases; the plasma level cannot reflect the local environment. Therefore, whether these inflammatory proteins with differences in plasma have different expression levels in intestinal tissues needs further study. Second, this study is a cross-sectional study, which cannot explain the causal relationship between diseases and inflammatory proteins changes. In order to clarify the pathophysiological role of inflammatory factors in NEC, it is necessary to compare them in infants before and after the onset of NEC.

In summary, our study found that 11 biomarkers (such as IL-8, IL-24, CCL20, OPG, TSLP, TRAIL, MMP-10, CXCL1, MCP-4, TNFSF14, and LIF) have high values in identifying NEC and determining the severity of NEC. Although the sensitivity and specificity of individual factors are not high enough, good diagnostic or predictive ability can be obtained by combining these biomarkers. The combination of IL-8, IL-24 and CCL20 has the best value to screen the preterm infants at early stage with high risk of NEC. The results of this study provide new strategy for early detection NEC.

## Supplementary Information

Below is the link to the electronic supplementary material.

Supplementary file1 (DOCX 330 KB)

## Data Availability

All data pertaining to this study are available upon justified request through the author in correspondence.
