# Supplementary material for: Screening inflammatory protein biomarkers on premature infants with necrotizing enterocolitis
Source: Inflamm Res. 2023 Feb 18;72(4):757–68. doi: 10.1007/s00011-023-01702-6 (PMC10129932; doi:10.1007/s00011-023-01702-6)
Supplement: Supplementary file 1 — Supplementary file1 (DOCX 330 KB) [file 11_2023_1702_MOESM1_ESM.docx]

**Screening inflammatory protein biomarkers on premature infants with necrotizing enterocolitis**

**Running title:** Inflammatory biomarkers on preterm NEC infants

Huifang Dong MD^1, 2*^, Lingling Zhang MSc^1*^, Bingbing Li MD^1^, Jing Li MD^2^, Yanshan Chen MD^2^, Seidu R. Richard MD, PhD^1^，Yiran Xu MD, PhD^1^, Changlian Zhu, MD, PhD^1,3,4^

**Journal：**Inflammation Research

**Corresponding authors:** Yiran Xu, Henan Key Laboratory of Child Brain Injury, Third Affiliated Hospital, Zhengzhou 450052, China. Tel: +86 371 6690 3550, Email: [yiran.xu@zzuneuro.cn](mailto:yiran.xu@zzuneuro.cn) or Changlian Zhu, Center for Brain Repair and Rehabilitation, Institute of Neuroscience and Physiology, University of Gothenburg, Gothenburg 40530, Sweden. Tel: +46 31 786 3339, Email: [changlian.zhu@neuro.gu.se](mailto:changlian.zhu@neuro.gu.se) or Third Affiliated Hospital of Zhengzhou University, Zhengzhou 450052, China. Tel: +86 371 6690 3974 Email: [zhuc@zzu.edu.cn](mailto:zhuc@zzu.edu.cn)

**Supplementary Materials**

**Supplementary Table1. Proinflammatory biomarkers between NEC and HC group**

| Biomarkers（NPX） | NEC vs. HC group(n) | Univariate analysis | | Multivariate Analysis^#^ | |
| --- | --- | --- | --- | --- | --- |
|  |  | OR (95%CI) | *p* value | OR (95%CI) | *p* value |
| IL-8 | 30/29 | 5.897(2.264-15.361) | **<0.001***** | 7.50(2.235-25.171) | **0.001**** |
| OPG | 30/29 | 18.978(3.651-98.660) | **<0.001***** | 32.410(4.270-246.003) | **0.001**** |
| TRAIL | 30/29 | 0.115(0.033-0.406) | **0.001**** | 0.117 (0.033-0.413) | **0.001**** |
| CXCL1 | 30/29 | 2.977(1.598-5.547) | **0.001**** | 3.295(1.610-6.745) | **0.001**** |
| TSLP | 30/29 | 6.443(2.132-19.469) | **0.001**** | 15.728(3.369-73.413) | **<0.001***** |
| MCP-4 | 30/29 | 0.340(0.166-0.698) | **0.003**** | 0.262(0.111-0.617) | **<0.001***** |
| TNFSF14 | 30/29 | 1.893(1.110-3.231) | **0.019*** | 1.193(1.078-3.395) | **0.027*** |
| IL-24 | 30/29 | 2.60(1.568-4.309) | **<0.001***** | 3.320(1.734-6.358) | **<0.001***** |
| MMP-10 | 30/29 | 11.773(2.805-49.408) | **0.001**** | 17.433(2.999-101.330) | **0.001**** |
| LIF | 30/29 | 9.353(2.50-34.992) | **0.001**** | 8.499(2.091-34.553) | **0.003**** |
| CCL20 | 30/29 | 3.524(1.858-6685) | **<0.001***** | 5.020(2.180-11.560) | **<0.001***** |

#: Adjusted by gestational age, birth weight, sex, and fetal distress. **p*<0.05, ***p*<0.01, ****p*<0.001.

**Supplementary Table2. Proinflammatory biomarkers between NEC and sepsis group**

| Biomarkers（NPX） | NEC vs Sepsis group(n) | Univariate analysis | | Multivariate analysis^#^ | |
| --- | --- | --- | --- | --- | --- |
|  |  | OR (95%CI) | *p* value | OR (95%CI) | *p* value |
| IL-8 | 30/29 | 1.736(1.195-2.521) | **0.004**** | 1.802(1.221-2.659) | **0.003**** |
| OPG | 30/29 | 2.660(1.306-5.416) | **0.007**** | 2.838(1.333-6.042) | **0.007**** |
| TRAIL | 30/29 | 0.318(0.150-0.673) | **0.003**** | 0.298(0.137-0.649) | **0.002**** |
| CXCL1 | 30/29 | 2.841(1.504-5.368) | **0.001**** | 2.754(1.437-5.278) | **0.002**** |
| TSLP | 30/29 | 3.730(1.484-9.373) | **0.005**** | 3.90(1.495-10.178) | **0.005**** |
| MCP-4 | 30/29 | 0.041(0.202-0.798) | **0.009**** | 0.388(0.188-0.799) | **0.010*** |
| TNFSF14 | 30/29 | 2.381(1.214-4.670) | **0.012*** | 2.277(1.128-4.597) | **0.022*** |
| IL-24 | 30/29 | 2.148(1.353-3.412) | **0.001**** | 2.333(1.413-3.853) | **0.001**** |
| MMP-10 | 30/29 | 3.694(1.461-9.341) | **0.006**** | 4.743(1.585-14.195) | **0.005**** |
| LIF | 30/29 | 2.499(1.286-4.857) | **0.007**** | 2.670(1.224-5.823) | **0.014*** |
| CCL20 | 30/29 | 1.631(1.113-2.389) | **0.012*** | 1.813(1.182-2.782) | **0.006**** |

# Adjusted by gestational age, birth weight, sex, and fetal distress. **p*<0.05, ***p*<0.01.

**Supplementary Fig.1. Diagnostic value of the biomarkers between NEC and HC group**


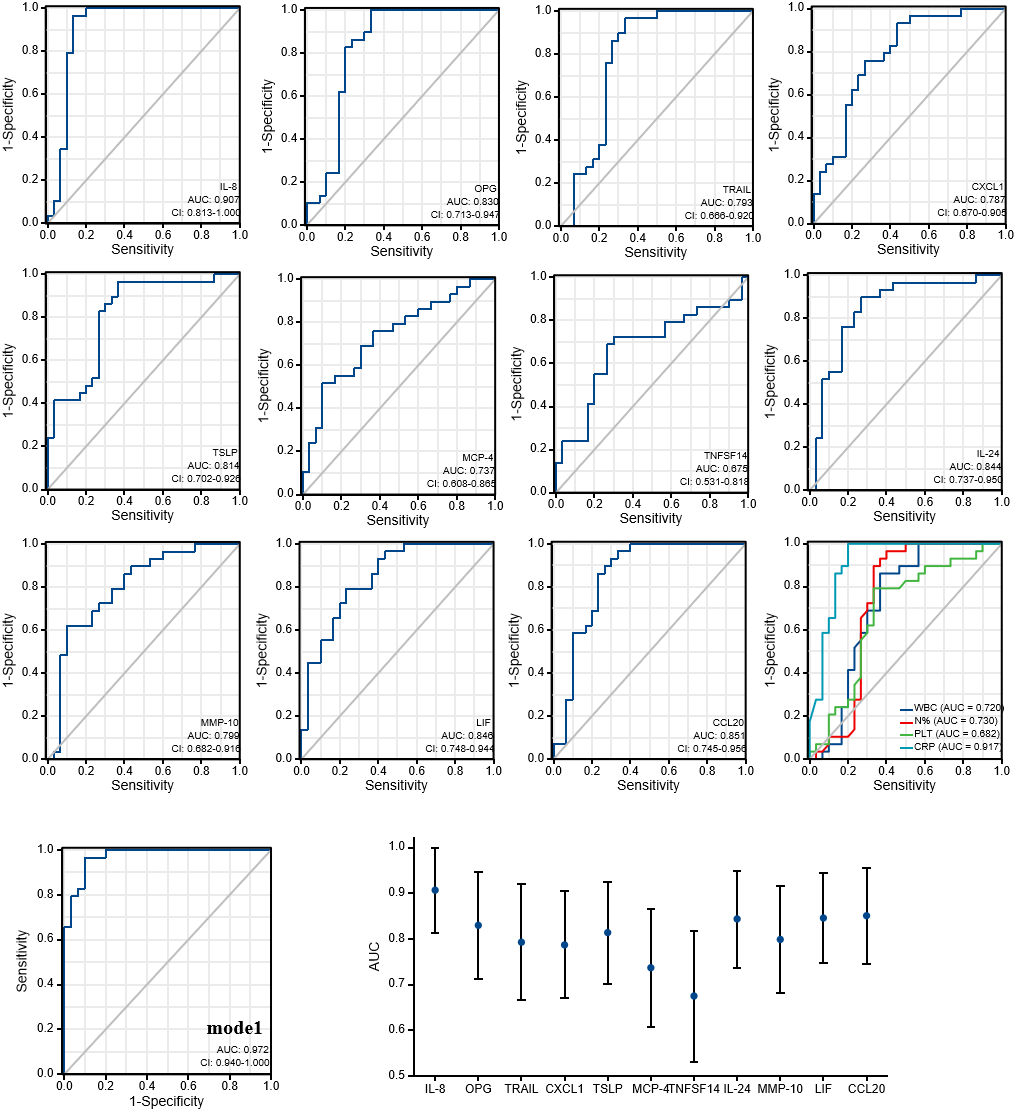


Diagnostic value of the biomarkers between NEC and HC group: AUC for different biomarkers. AUC 0.5-0.7: acceptable; AUC 0.7-0.85: good; AUC 0.85-0.95: excellent. Model 1: the11 markers are combined.

**Supplementary Fig.2. Diagnostic value of the biomarkers between NEC and sepsis group**


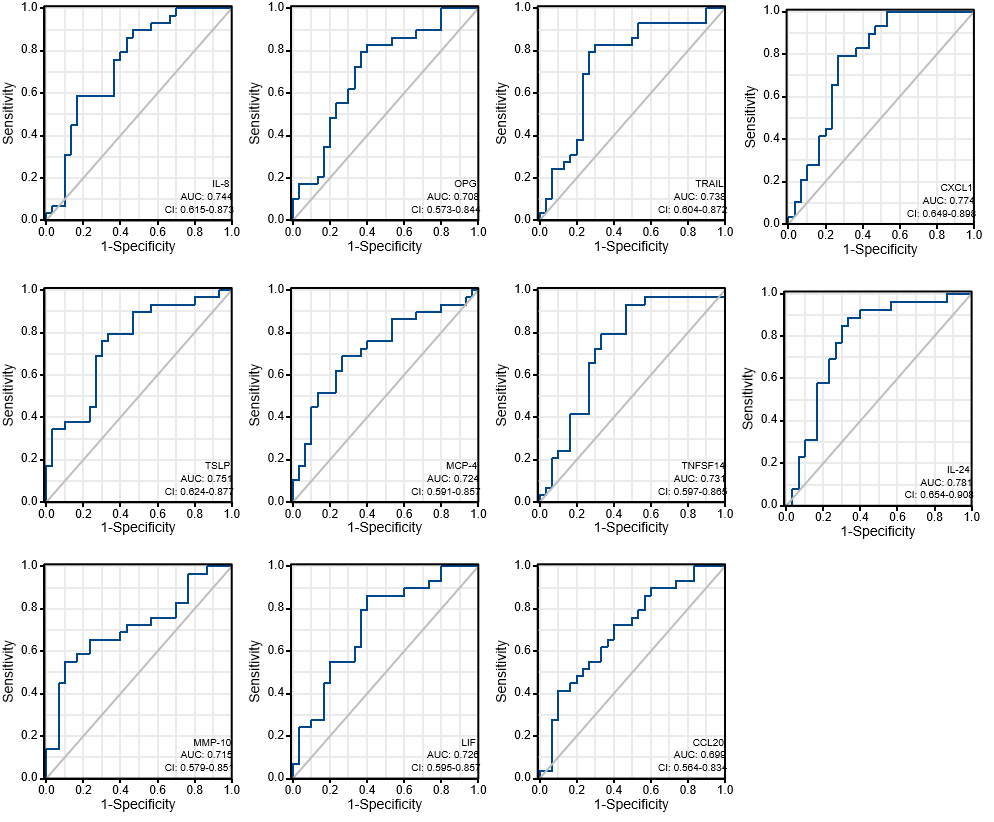


Diagnostic value of the biomarkers between NEC and Sepsis group: AUC for different biomarkers. AUC 0.5-0.7: acceptable; AUC 0.7-0.85: good; AUC 0.85-0.95: excellent.

**Supplementary Fig.3. Diagnostic value of the biomarkers between NEC stage II and III**


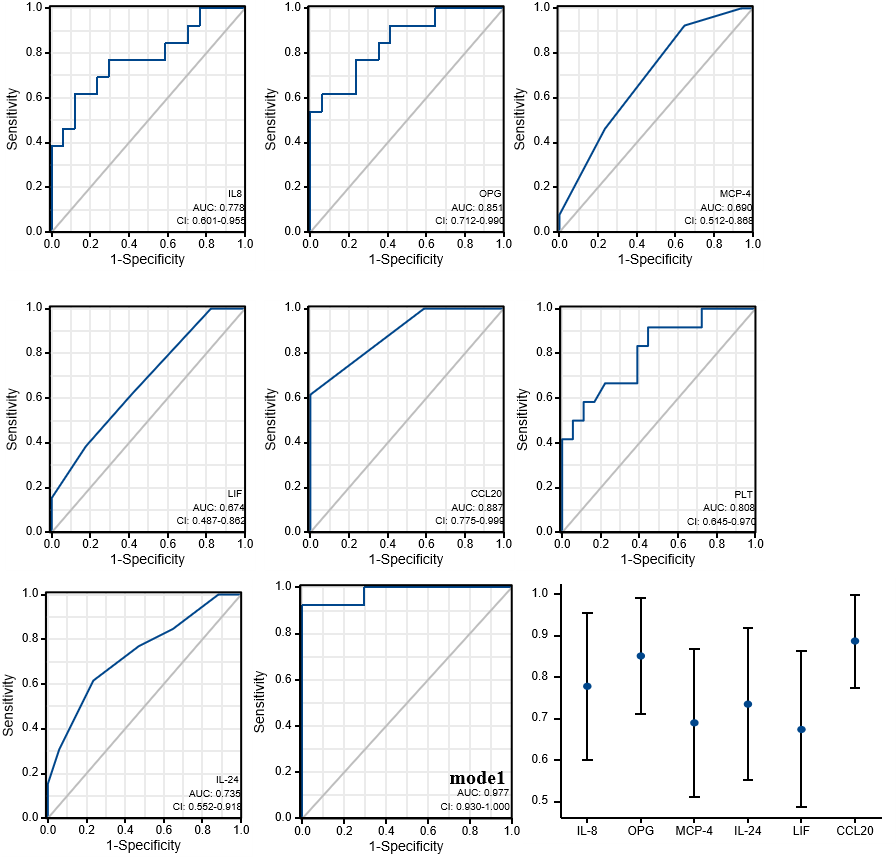


Diagnostic value of the biomarkers between NE C stage II and III groups: AUC for different biomarkers. AUC 0.5-0.7: acceptable; AUC 0.7-0.85: good; AUC 0.85-0.95: excellent. Model 1：the 6 markers are combined.
